# Computational prediction of MoRFs based on protein sequences and minimax probability machine

**DOI:** 10.1186/s12859-019-3111-z

**Published:** 2019-10-28

**Authors:** Hao He, Jiaxiang Zhao, Guiling Sun

**Affiliations:** College of Electronic Information and Optical Engineering, Nankai University, Tianjin, China

**Keywords:** Molecular recognition features, Intrinsically disordered proteins, Minimax probability machine

## Abstract

**Background:**

Molecular recognition features (MoRFs) are one important type of disordered segments that can promote specific protein-protein interactions. They are located within longer intrinsically disordered regions (IDRs), and undergo disorder-to-order transitions upon binding to their interaction partners. The functional importance of MoRFs and the limitation of experimental identification make it necessary to predict MoRFs accurately with computational methods.

**Results:**

In this study, a new sequence-based method, named as MoRF_MPM_, is proposed for predicting MoRFs. MoRF_MPM_ uses minimax probability machine (MPM) to predict MoRFs based on 16 features and 3 different windows, which neither relying on other predictors nor calculating the properties of the surrounding regions of MoRFs separately. Comparing with ANCHOR, MoRFpred and MoRF_CHiBi_ on the same test sets, MoRF_MPM_ not only obtains higher AUC, but also obtains higher TPR at low FPR.

**Conclusions:**

The features used in MoRF_MPM_ can effectively predict MoRFs, especially after preprocessing. Besides, MoRF_MPM_ uses a linear classification algorithm and does not rely on results of other predictors which makes it accessible and repeatable.

## Background

Intrinsically disordered proteins (IDPs) are protein sequences that contain at least one region lacking a unique 3-D structure [1]. Although not being folded, IDPs perform a variety of important functions such as molecular recognition, transport catalysis, signaling regulation, entropic chain activities, and so on [2]. Furthermore, a single protein may contain several disordered regions that possess different functions [3]. The functions of disordered regions usually stem from their ability to bind to partner molecules [4]. Disordered regions can provide malleable interfaces which can recognize molecules through increase complementarity via induced fit or offer alternative interaction upon variable conditions and more complex cellular responses [5]. These recognition regions may form folded and complementary interfaces, while the neighboring regions, often denoted as fuzzy, can maintain their disordered state [6]. The notion of fuzziness implies that conformational heterogeneity can be maintained upon interactions of IDPs [7]. The disordered regions mainly contain two types of binding motifs: short linear motifs (SLiMs) and MoRFs. SLiMs are enriched in IDRs. They are generally conserved and 3-10 residues long, and thus may not fall into regular secondary structures [7]. MoRFs generally locate within longer IDRs and are up to 70 residues long [8]. They promote specific protein-protein interactions, and undergo disorder-to-order transitions upon binding their partners [4]. According to the structures they adopt in bound state, MoRFs can be classified into four subtypes: α-MoRFs, β-MoRFs, ι-MoRFs and complex-MoRFs [9]. The first three types form α-helix, β-strand, irregular secondary structure and the last one contains multiple secondary structures when bound [9].

Because of the functional importance of MoRFs and the limitation of experimental identification, several computational methods have been produced in recent years, such as *α*-MoRF-Pred I [10], *α*-MoRF-PredII [11], ANCHOR [12, 13], MoRFpred [14], MSPSSMpred [15] and MoRF_CHiBi_ [16]. *α*-MoRF-PredII is an improved method for *α*-MoRF-Pred I, which is limited to predict *α*-MoRFs. ANCHOR and MoRFpred are the most used comparison methods in recent years. ANCHOR is a web based method, which predicts protein binding regions that are disordered in isolation but can undergo disorder-to-order transition upon binding by using the energy estimation approach of IUPred [17]. MoRFpred is also a web based method, which is a comprehensive method. It calculates a MoRF propensity score using a linear kernel support vector machine (SVM) based on nine sets of features: physicochemical properties in Amino Acid Index [18], Position Specific Scoring Matrices (PSSM), predicted relative solvent accessibility [19], predicted B-factors [20] and the results of five different intrinsic disorder predictors. Then, using PSI-BLAST [21] to align the input sequence with the training sequence to gain an alignment e-value, which is used to adjust the calculated MoRF propensity score. MSPSSMpred using a radial basis function (RBF) kernel SVM model to predict MoRFs based on calculated conservation scores. This method does not use predicted results from other predictors as input, and the performance in AUC is approximate to MoRFpred. MoRF_CHiBi_ uses two SVM models to predict MoRFs based on physicochemical properties of amino acids. The first model use a sigmoid kernel SVM to predict MoRF propensities, which target direct similarities between MoRF sequences. The second model focus on the general contrast of amino acid composition of MoRFs, Flanks and the general protein population using a RBF Gaussian kernel SVM. Finally, join the results of the two SVM models and compute the propensity score using Bayes rule. MoRF_CHiBi_ is a very good MoRF predictor that does not rely on other predictors.

In this paper, we propose a novel sequence-based method, MoRF_MPM_, for predicting MoRFs. First, simulated annealing algorithm is utilized for selecting candidate feature sets from Amino Acid Index (AA Index) [18]. Then, five structural features from our previous study [22] about IDPs prediction are put into candidate sets for further selection, which contain Shannon entropy and topological entropy calculated directly from protein sequences, as well as three amino acid propensities from GlobPlot NAR paper [23]. Finally, we select 16 features and 3 different windows to preprocess the protein sequences and use MPM [24] which is a linear classification algorithm to predict MoRFs. The simulation results show that even though MoRF_MPM_ just uses 16 features, 3 different windows and a linear classification, it obtains higher AUC and TPR than ANCHOR, MoRFpred and MoRF_CHiBi_.

## Results

### Datasets

In order to compare our method with ANCHOR, MoRFpred and MoRF_CHiBi_, we use the datasets collected by Disfani et al. [14], which are also used to train and test MoRFpred and MoRF_CHiBi_. Disfani et al. collected a lot of protein complexes concerning interactions of protein-peptide from Protein Data Bank (PDB) [25] of March 2008 and filtered them on several principles to identify peptide regions of 5 to 25 residues which were presumed to be MoRFs. The obtained 840 protein sequences are divided into a training set (TRAINING) and a test set (TEST). There are 181 helical, 34 strand, 595 coil and 30 complex MoRF regions on the two sets. TRAINING contains 421 sequences which consists of 245,984 residues with 5396 MoRF residues. TEST contains 419 sequences which consists of 258,829 residues with 5153 MoRF residues. Besides, using the same protocol [26, 27], they also collected TESTNEW set from PDB entries deposited between January 1 and March 11, 2012. TEST2012 contains 45 sequences which consists of 37,533 residues with 626 MoRF residues. In addition, we use the EXP53 collected by Malhis et al. [28] as the third test set. The test set contains 53 non-redundant sequences possessing MoRFs, which are collected from four publicly available experimentally validated sets. EXP53 includes 2432 MoRF residues which consist of 729 residues from short MoRF regions (up to 30 residues) and 1703 residues from long MoRF regions (longer than 30 residues). For more intuitive description of the four datasets, Table 1 lists their specific information.

**Table 1:** Datasets used in this paper

	TRAINING	TEST	TESTNEW	EXP53
Number of Sequences	421	419	45	53
Number of MoRFs Residues	5396	5153	626	2432
Number of non-MoRFs Residues	240,588	253,676	36,907	22,754
Total Residues	245,984	258,829	37,533	25,186

The detail information of four datasets

### Performance evaluation

We use AUC to evaluate the performance of different candidate feature sets and different windows. It is also utilized to compare our method with other methods. AUC is the area under the ROC curve, which can provide an overall assessment about the prediction. In order to compare the performance of each method in detail, we also calculate ACC and FPR at different TPR. ACC describes the total number of residues that are correctly predicted, FPR is the false positive rate and TPR is the true positive rate. They are defined as:

1 $$ \mathrm{ACC}=\frac{TP+ TN}{N_{\mathrm{MoRF}}+{N}_{\mathrm{non}}},\kern0.5em \mathrm{FPR}=\frac{TN}{N_{\mathrm{non}}},\kern0.5em \mathrm{TPR}=\frac{TP}{N_{\mathrm{MoRF}}}, $$

Where *TP* and *TN* are the numbers of accurately predicted MoRFs residues and non-MoRFs residues, *N*_MoRF_ and *N*_non_ are the total numbers of MoRFs residues and non-MoRFs residues, respectively.

### Selecting the optimal feature set

**Fig. 1:**
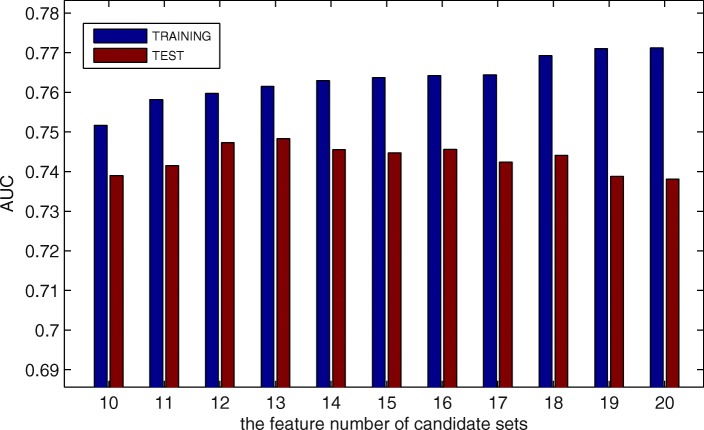
Predictive performance with different number of properties from AA Index. The blue line is the AUC values on TRAINING set, and the red line is the AUC values on TEST set

Firstly, we use simulated annealing algorithm to select several candidate sets of different feature number based on the TRAINING from 544 amino acid index. Then, we use MPM [24, 29] to predict MoRFs based on these candidate feature sets, and select the feature set with the best performance. Figure 1 shows the predictive results on TRAINING and TEST with different candidate feature sets. The blue line represents the AUC values on TRAINING, the red line represents the AUC values on TEST. The distances between AUC values on the two sets reflect the over-fitting situation of each candidate set, and the shorter the distance, the more robust the predictive performance. Because MPM is a linear classification algorithm, the over-fitting is not serious in all of these candidate sets. However, it is obvious that when the feature number in the candidate set is 12 or 13, the predictor gains more robust performance and better AUC value on TEST at the same time.

**Fig. 2:**
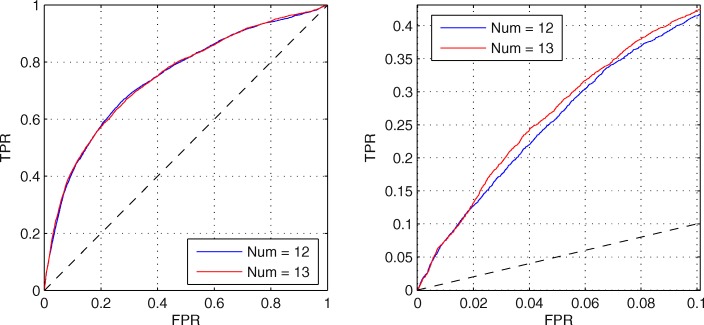
ROC curves when the feature numbers in candidate sets are 12 and 13. The left figure is the full ROV curves. The right figure is the ROC curves at low FPR

When the feature number of candidate set is 12 or 13, the predictive performance is approximate. Thus, to further compare their performance, the ROC curves are shown on Fig. 2. The left one shows the full ROC curves of them, which almost overlap. Since we are more concerned about the predictive performance at low FPR, the right figure shows the ROC curves at FPR < 0.1. Obviously, in this area, predictive performance on 13 is much better. Thus, we select the candidate set with 13 features as the final candidate feature set from AA Index, which is listed with the AA Index accession numbers in Table 2.

**Table 2:** AA Index accession numbers of selected features

CIDH920101	ROBB760101	CORJ870103	MIYS990104
EISD860103	ROBB760108	CORJ870106	–
NISK860101	ROBB760112	CORJ870107	–
QIAN880105	ROBB760113	CORJ870108	–

These 13 features are collected by simulated annealing algorithm from AA Index

**Fig. 3:**
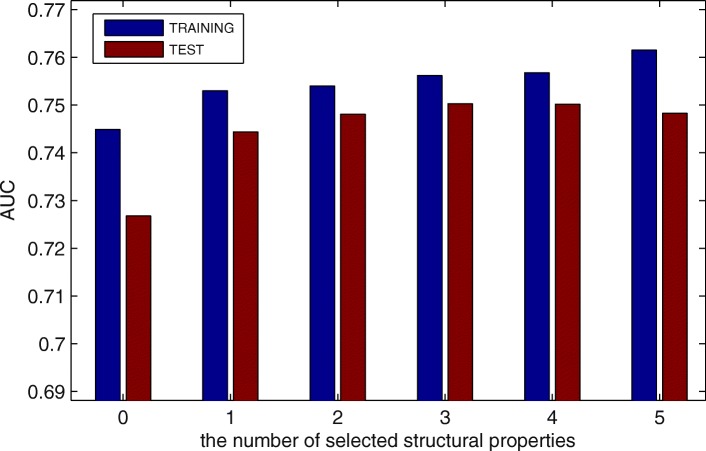
Predictive performance with different number of structural properties. The blue line is the AUC values on TRAINING set, and the red line is the AUC values on TEST set

**Fig. 4:**
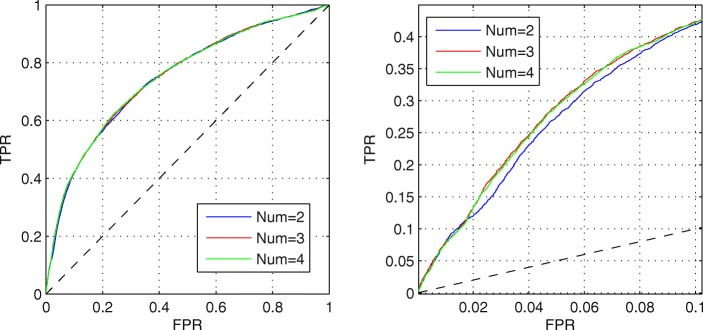
ROC curves with different number of structural properties. The left figure is the full ROV curves. The right figure is the ROC curves at low FPR

After that, we put the five structural properties which selected by our previous study [22] about IDPs prediction into the candidate feature set. Then, we change the number of structural properties in the candidate feature set and use MPM to predict MoRFs. Since there are only five structural features in total, we use the enumeration method to select structural properties for each candidate feature set with different number of structural properties. Figure 3 shows the best AUC values with different numbers of structural properties. Obviously, when the number is between 2 and 4, the performance is similar and obviously better than other cases. To further compare their performance, the ROC curves are shown on Fig. 4. Though the full ROC curves of them almost overlap as shown in the left figure, 3 and 4 obtain better performance at FPR < 0.1 as shown in the right figure. Considering that the AUC value of 3 is slightly higher than that of 4 on TEST set, we finally select the three structural properties which contain topological entropy calculated directly from protein sequences, as well as the Remark 465 and Deleage/Roux propensities from GlobPlot NAR paper [23].

### Selecting the appropriate windows sizes

**Fig. 5:**
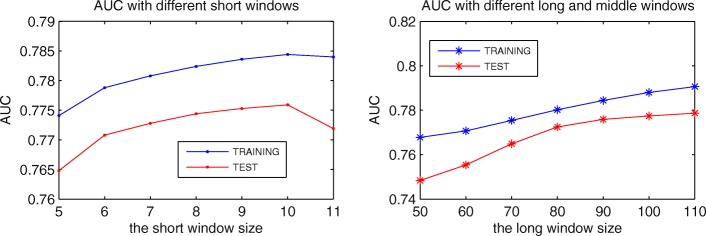
Predictive performance with different windows sizes. The left figure is the AUC values with different short windows. The right figure is the AUC values with different long and middle windows. The size of the middle window is always the half size of the long window

We select three windows to preprocess protein sequences. Based on each window, we calculate the 16 selected features. Thus, each residue can obtain a 48 dimensional feature vector. Then, we change the sizes of three windows, and use MPM to predict MoRFs. The appropriate size of three windows are set by comparing their predictive performance on TRAINING and TEST. Figure 5 shows the predictive performance with different windows sizes. The middle window is always set to the half size of the long window. In the left figure, we fix the size of the long and middle window to 90 and 45, and change the size of the short window from 5 to 11. Obviously, when the short window is set to 10, the AUC is better on TEST set.

**Fig. 6:**
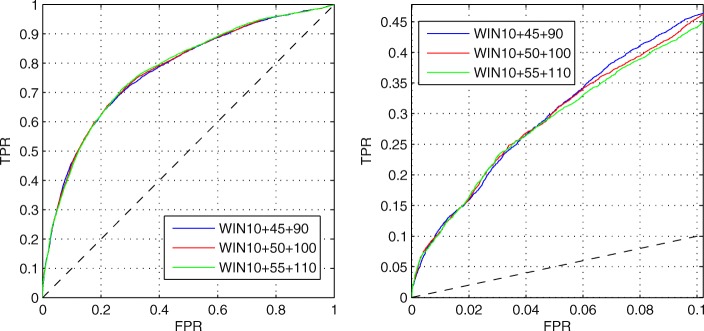
ROC curves for with different long and middle windows. The short window is set to 10. The left figure is the full ROV curves. The right figure is the ROC curves at low FPR

Then, we fix the short window to 10 and change the size of the long and middle windows as shown in the right figure of Fig. 4. The long window size is varied from 50 to 110, and the middle window size is changed following the long window. At the beginning, as the long window size increases, the AUC of both data sets increases, and the distance between them decreases. But when the size is larger than 80, the AUC of the two data sets grows slowly, and the distance between them increases. Moreover, when the size is larger than 90, the AUC of TEST tends to be stable. Figure 6 shows the ROC curves on TEST set with the long window size between 90 and 110. In the left figure, the ROC curves of the three sizes almost overlap. However, the ROC curve of 90 is better at low FPR as shown in the right figure. Considering that the proportion of MoRF residues is only about 2% in the TRAINING and TEST sets, we pay more attention to the predictive performance at low FPR. Thus, the long and middle windows are eventually set to 90 and 45.

Considering that researchers may require different precision depending on the applications, we do not set a standard threshold value. However, if one needs a binary categorical prediction, Table 3 provides three threshold values and their predictive results for reference, according to the FPRs on TRAINGING set. The threshold value can be selected in (− 0.5, 0.5), and the larger the value is, the larger the FPR.

**Table 3:** Three threshold values and their predictive results

TRAINING_FPRs	FPR = 0.05		FPR = 0.1		FPR = 0.15	
Thresholds	−0.12		− 0.0735		−0.0438	
	TPR	FPR	TPR	FPR	TPR	FPR
TEST	0.313	0.052	0.458	0.098	0.535	0.141
TESTNEW	0.300	0.037	0.401	0.074	0.470	0.116

The thresholds are calculated by the fixed FPR values on TRAINING set. The default value of the threshold is 0

### Comparing with other prediction methods

In this part, we compare our method MoRF_MPM_ with ANCHOR, MoRFpred and MoRF_CHiBi_ for three test sets TEST, TESTNEW and EXP53. The results of other methods on these three sets are adopted from [16, 28]. Table 4 shows the AUC values for the four methods on TEST and TESTNEW sets. Obviously, MoRF_MPM_ achieves higher AUC than ANCHOR, MoRFpred and MoRF_CHiB_ on both TEST and TESTNEW sets.

**Table 4:** AUC on TEST and TESTNEW sets

	MoRF_MPM_	MoRF_CHiBi_	MoRFpred	ANCHOR
TEST	0.777	0.746	0.673	0.600
TESTNEW	0.790	0.770	0.697	0.638

The AUC values of four methods on TEST and TESTNEW sets

On TEST set, we also compare ACC and FPR at different TPR with other methods, as shown in Table 5. MoRF_MPM_ achieves the lower FPRs and higher ACCs on the three TPRs compared with ANCHOR, MoRFpred and MoRF_CHiBi_. In other words, MoRF_MPM_ can obtain higher TPR at low FPR.

**Table 5:** ACC and FPR at different TPR on TEST set

	TPR = 0.222		TPR = 0.254		TPR = 0.389	
	FPR	ACC	FPR	ACC	FPR	ACC
MoRF_MPM_	0.030	0.955	0.038	0.948	0.072	0.917
MoRF_CHiBi_	0.035	0.951	0.045	0.942	0.098	0.893
MoRFpred	0.037	0.948	0.049	0.937	0.137	0.854
ANCHOR	0.092	0.894	0.125	0.863	0.253	0.740

FPR and ACC as functions of TPR are calculated on TEST set

In addition, Table 6 shows the AUC results of these four methods on EXP53 set. In EXP53_short set, only MoRF regions with up to 30 residues are considered, while longer MoRF regions are masked out. In EXP53_long set, only MoRF regions longer than 30 residues are considered, while shorter MoRF regions are masked out [28]. From Table 6, MoRF_MPM_ also obtains higher AUC on EXP53_all, EXP53_short and EXP53_long sets.

**Table 6:** AUC on EXP53 set

	MoRF_MPM_	MoRF_CHiBi_	MoRFpred	ANCHOR
EXP53_all	0.761	0.714	0.620	0.615
EXP53_short	0.814	0.790	0.673	0.683
EXP53_long	0.739	0.681	0.598	0.586

The AUC values of four methods on EXP53_all, EXP53_short and EXP53_long sets

## Discussion

We propose a new method, MoRF_MPM_, to predict MoRFs within protein sequences. It uses MPM to train the predictor based on 16 features and 3 different windows. The feature set contains 13 physicochemical properties selected from Amino Acid Index and 3 structural properties selected from our previous study [22] about IDPs prediction including topological entropy and two amino acid propensities in GlobPlot NAR paper [23]. We compare MoRF_MPM_ with ANCHOR, MoRFpred and MoRF_CHiBi_ on three different test sets: TEST, TESTNEW and EXP53. The results show that MoRFMPM obtains better performance on these test sets.

**Fig. 7:**
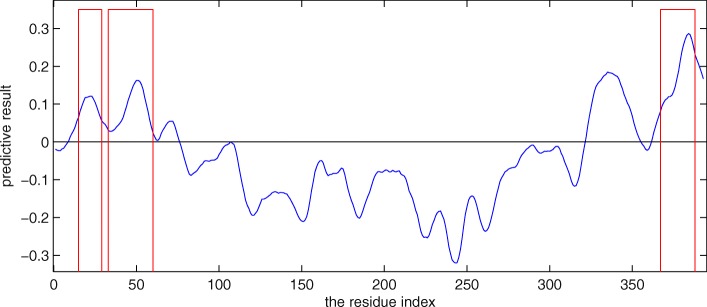
Predictive results for the protein p53. The blue line is the predictive results of our method. The red lines indicate confirmed MoRFs. The threshold is 0, which is shown as the black line. If the regions in blue line are higher than the black line, they are predicted to be MoRFs

To further illustrate the predictive performance of MoRF_MPM_, the protein p53 is predicted as an example, as shown in Fig. 7. The protein p53 is a master protein in tumor regulation, which is one of the most extensively studied IDPs [30, 31]. The N-terminal and C-terminal regions of this protein are confirmed to contain MoRFs [32–34] which are enclosed by the red lines in Fig. 7. The blue line is the predictive results of MoRF_MPM_ for each residue. From Fig. 7, MoRF_MPM_ can effectively identify MoRFs of the protein p53.

The following points enable MoRF_MPM_ to achieve such good performance. First, the appropriate preprocessing highlights the relationship between the residue and its surrounding residues. Second, the feature set used in MoRF_MPM_ is highly effective for predicting MoRFs, especially after preprocessing. Third, instead of considering the properties of Flanks with fix length, MoRF_MPM_ uses a long window of 90 to describe the influence of adjacent areas on MoRFs, and uses a short window of 10 to highlight the properties of MoRFs. Though the long window may contain much non-MoRFs information when calculating the feature vector of MoRF residues, MoRF_MPM_ uses a middle window of 45 to reduce the noise brought by the long window. Finally, although MPM is a linear classification algorithm, it is efficient and robust, especially when there are not too many features used.

## Conclusions

In this paper, a new sequence-based method, named as MoRF_MPM_, is proposed to predict MoRFs. MoRF_MPM_ calculate 16 features for each residue through preprocessing with 3 different windows, and use MPM to predict MoRFs. MoRF_MPM_ does not depend on results of other predictors. Comparing with ANCHOR, MoRFpred and MoRF_CHiBi_ on three different test sets: TEST, TESTNEW and EXP53, MoRF_MPM_ obtains the best AUC on these test sets. In addition, on TEST set, MoRF_MPM_ achieves lower FPR and higher ACC when TPR is set to 0.222, 0.254 and 0.389. The predicting code of MoRF_MPM_ are available at https://github.com/HHJHgithub/MoRFs_MPM, where we also provide an example with the protein p53.

## Methods

### Preprocessing

To highlight the interrelation between residues, the protein sequences are preprocessed. For a general protein sequence *w* with length *L*, we select a window with the length of *N*(*N* < *L*) and fill *N*_0_ = ⌊(*N* − 1)/2⌋ zeros at the beginning and end of the sequence. Then we slide the window to intercept regions of length *N* successively with step of length 1. At this point, the sequence length becomes *L*_0_ = *L* + 2*N*_0_, and the intercepted region can be denoted as:

2 $$ {w}_i={w}_0(i)\cdots {w}_0\left(i+N-1\right),\kern1.25em 1\le i\le {L}_0-N+1\kern0.5em , $$

where *w*_0_ represents the sequence after zero-padding. For each *w*_*i*_, the values corresponding to the selected features are calculated as following:

3 $$ {\mathbf{v}}_i={\left[{M}_1\left({w}_i\right)\ {M}_2\left({w}_i\right)\cdots \kern0.5em {M}_k\left({w}_i\right)\cdots \right]}^{\mathrm{T}}\kern0.5em ,\kern1.25em 1\le i\le {L}_0-N+1. $$

*M*_*k*_(*w*_*i*_) denotes the value of *k*-th feature calculated on *w*_*i*_. For one amino acid property, *M*_*k*_(*w*_*i*_) denotes the average value of *w*_*i*_ mapped by the scale of the property. For Shannon entropy or topological entropy, *M*_*k*_(*w*_*i*_) denotes the value calculated on *w*_*i*_ by their respective formulas [22]. After that, we assign **v**_***i***_ to each residue in *w*_*i*_. For each residue, add up all **v**_***i***_ of them and divide by their respective cumulative number. The feature vector **x**_*j*_ (1 ≤ *j* ≤ *L*) of each residue can be expressed as:

4 $$ {\mathbf{x}}_j=\left\{\begin{array}{c}\frac{1}{j+{N}_0}\sum \limits_{i=1}^{j+{N}_0}{\mathbf{v}}_{\boldsymbol{i}}\kern0.5em ,\kern1em 1\le j\le {N}_0\\ {}\frac{1}{N}\sum \limits_{i=j+{N}_0-N+1}^{j+{N}_0}{\mathbf{v}}_{\boldsymbol{i}}\kern0.5em ,\kern1em {N}_0<j\le L-{N}_0\\ {}\frac{1}{L_0-j-{N}_0+1}\sum \limits_{i=j+{N}_0-N+1}^{L_0-N+1}{\mathbf{v}}_{\boldsymbol{i}}\kern0.5em ,\kern0.75em L-{N}_0<j\le L\end{array}\right. $$

### Feature selection

**Fig. 8:**
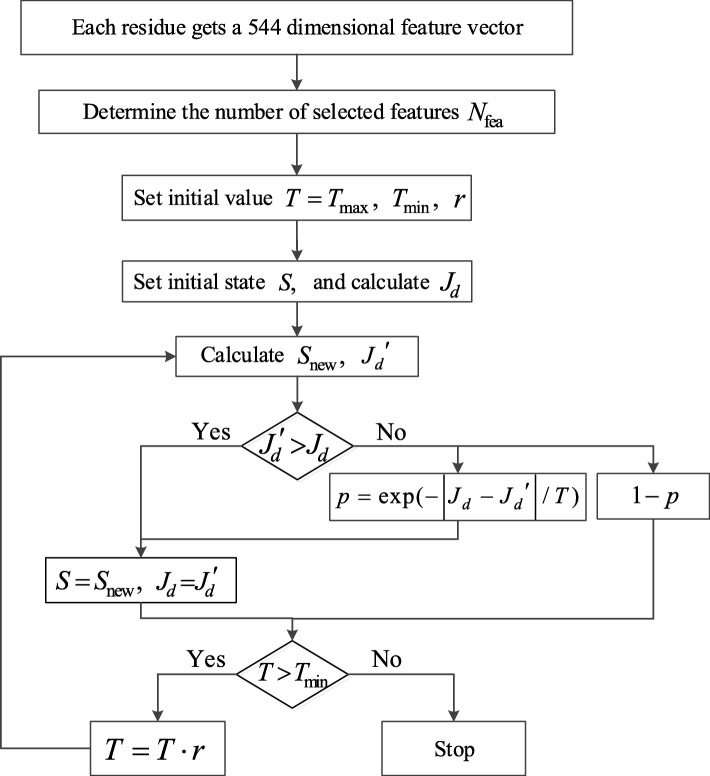
The process of feature selection by simulated annealing algorithm. Using simulated annealing algorithm, we select properties from AA Index

As mentioned, our feature set contains two parts: properties from AA Index [18] and structural properties. We first select properties from AA Index using simulated annealing algorithm, as shown in Fig. 8.

The detailed steps are as follows:

1. According to the section of preprocessing, the sequences in TRAINING set are preprocessed based on the 544 amino acid scales from AA Index. Then, each residue can obtain a 544 dimensional feature vector.
2. Set the number of selected features *N*_fea_.
3. Set the initial temperature *T* = *T*_*max*_, the lower limit temperature *T*_*min*_ and the annealing rate *r*.
4. *N*_*fea*_ features are selected randomly from 544 scales as the initial state *S*. Then, the distance between MoRF residues and non-MoRF residues is denoted as *J*_*d*_ and calculated using the selected *N*_*fea*_ feature vector. *J*_*d*_ can be expressed by *J*_*d*_ = tr(**S**_*w*_ + **S**_*b*_), where **S**_*b*_ denotes the between-class scatter matrix ${\mathbf{S}}_b=\sum \limits_{i=1}^2{P}_i\left({\mathbf{m}}_i-\mathbf{m}\right){\left({\mathbf{m}}_i-\mathbf{m}\right)}^T$ and **S**_*w*_ is the within-class scatter matrix ${\mathbf{S}}_w=\sum \limits_{i=1}^2{P}_i\frac{1}{N_i}\sum \limits_{j=1,\kern0.5em {x}_j\in {X}_i}^{N_i}\left({\mathbf{x}}_j-{\mathbf{m}}_i\right){\left({\mathbf{x}}_j-{\mathbf{m}}_i\right)}^T$. Besides, **m**_*i*_ represents the mean vector of the *i*-th class and **m** represents the total mean vector. Thus, the larger *J*_*d*_ is, the more separable the two types of samples are.
5. Randomly select a feature that does not belong to state *S* from 544 scales, and make it replace any one of *S* to form a new state *S*_*new*_. Calculate the distance ${J}_d^{\prime }$ in the new state.
6. If ${J}_d^{\prime }>{J}_d$, go to (7). Otherwise, calculate $p=\exp \left(-\left|{J}_d-{J}_d^{\prime}\right|/T\right)$, then go to (7) with probability *p* and go to (8) with probability 1 − *p*.
7. Set *S* = *S*_*new*_, $$ {J}_d={J}_d^{\prime } $$.
8. If *T* > *T*_*min*_, set *T* = *T* ∙ *r* and go to (5). Otherwise, stop iteration.

In this paper, we set *T*_*max*_ = 1, *T*_*min*_ = 0.0001, *r* = 0.9995. The parameter *N*_fea_ is set from 10 to 20, and thus we obtain 11 candidate feature sets. Then, we use the 11 candidate feature sets to train MPM respectively, and select the feature set with the best prediction performance.

In addition, we select structure properties from five features used by our previous research [22] about IDPs prediction which contain Shannon entropy, topological entropy and three propensities from GlobPlot NAR paper [23] (http://globplot.embl.de/html/propensities.html) including the Deleage/Roux, Remark 465 and Bfactor (2STD) propensities. From [22], it has been shown that these five features can effectively predict IDPs. In addition, MoRFs generally locate within longer IDRs. Thus, we add these five features to the feature set obtained from AA index for further selection.

Since MoRFs generally locate within longer IDRs, the protein sequences with MoRFs usually contain three types of residues: MoRF residues, residues flanking (Flanks) the MoRFs and general non-MoRF residues. In other words, the Flanks represent other disordered residues on both sides of MoRFs, and general non-MoRF residues represent the ordered residues in the sequence. The properties of the three types of residues are different from each other. Thus MSPSSMpred and MoRF_CHiBi_ calculate the properties of Flanks separately, and select 5 and 8 residues on both sides of MoRFs as Flanks respectively. However, the number of Flank residues in each MoRF region is different, and even the number on both sides of one MoRF region is also different. Therefore, instead of calculating the properties of Flanks separately, we consider the impact of Flanks by choosing three different windows. The first window is shorter to highlight the properties of MoRFs, and the second window is longer to highlight the influence of Flanks. The third window is between them to reduce the noise generated by the longer window. The short window is selected from 5 to 11. Meanwhile, since MoRFs generally locate within longer IDRs, we select the long window no less than 50. If the long window is very long, it may contain much non-MoRFs information when calculating the feature vectors of MoRF residues. These non-MoRFs information will reduce the predictive accuracy of MoRFs at low FPR that we are most concerned about, even if we have used a short window. Therefore, we select a middle window half the length of the long window to improve the performance at low FPR.

For selecting the optimum features from 544 amino acid indexes, we just use the short window and set the length to 10, firstly. Through preprocessing the TRAINING set, each residue gets a 544 × 1 feature vector. Then, using simulated annealing algorithm, we select several feature sets with different feature numbers as candidate feature sets. After that, we put the five structural properties into them, and predict MoRFs based on MPM algorithm with the short window of 10 and the long window of 50 to select the best feature set. Finally, we change the number of structural properties to further optimize the feature set.

### MPM prediction model

MPM is a machine learning method of statistical learning proposed by Lanckriet et al. [24]. The main idea is to analyze the upper bound of classification error rate and make it as small as possible. Given a feature matrix to be classified $\mathbf{X}=\left[{\mathbf{x}}_1,{\mathbf{x}}_2,\cdots, {\mathbf{x}}_{N_s}\ \right]$, where *N*_*s*_ denotes the number of samples and **x**_*j*_(1 ≤ *j* ≤ *N*_*s*_) denotes the feature vector of the *j*-th sample. Suppose that these samples are divided into two groups **X**_1_, **X**_2_ ∈ **X**, and **X**_1_~(**μ**_1_, **R**_1_), **X**_2_~(**μ**_2_, **R**_2_). MPM is expected to build a classification surface **W**^T^**X** = *b*, which make the upper bound of the classification error rate as small as possible. Make an assumption that the correct classification satisfies **W**^T^**X**_1_ > *b* for the first group and **W**^T^**X**_2_ < *b* for the second group. The classification error rate is *P*{**W**^T^**X**_1_ ≤ *b*} for the first group and *P*{**W**^T^**X**_2_ ≥ *b*} for the second group. Then the classification surface constructed by MPM should satisfy the following requirements:

5 $$ \min \left[ Sup\ P\left\{{\mathbf{W}}^{\mathrm{T}}{\mathbf{X}}_1\le b\right\}\right]\kern1.25em and\kern1em \mathit{\min}\left[ Sup\ P\left\{{\mathbf{W}}^{\mathrm{T}}{\mathbf{X}}_2\ge b\right\}\right]. $$

Through a series of solutions, the optimization problem becomes:

$$ \underset{\mathbf{W},b}{\max}\kern0.5em \kappa \kern18.75em $$

6 $$ s.t.\kern1.00em \frac{1}{\kappa}\ge \left(\sqrt{{\mathbf{W}}^{\mathrm{T}}{\mathbf{R}}_1\mathbf{W}}+\sqrt{{\mathbf{W}}^{\mathrm{T}}{\mathbf{R}}_2\mathbf{W}}\right),\kern0.5em {\mathbf{W}}^{\mathrm{T}}\left({\boldsymbol{\upmu}}_1-{\boldsymbol{\upmu}}_2\right)=\mathbf{1}. $$

Since *κ* is only an intermediate variable, the optimization problem can be expressed as:

7 $$ \underset{\mathbf{W}}{\min}\sqrt{{\mathbf{W}}^{\mathrm{T}}{\mathbf{R}}_1\mathbf{W}}+\sqrt{{\mathbf{W}}^{\mathrm{T}}{\mathbf{R}}_2\mathbf{W}}\kern1.75em s.t.\kern0.75em {\mathbf{W}}^{\mathrm{T}}\left({\boldsymbol{\upmu}}_1-{\boldsymbol{\upmu}}_2\right)=\mathbf{1}. $$

The classification surface of MPM is finally reduced to solution formula Eq.7. It is a second order cone program problem, which can be solved by iterative least square method and interior point method. In this paper, we use the iterative least square method given in the reference [29]. Assuming that **W**_∗_ is the calculated optimal value, then the optimal *κ* and *b* can calculated by:

8 $$ {\kappa}_{\ast }=\frac{1}{\left(\sqrt{{{\mathbf{W}}_{\ast}}^{\mathrm{T}}{\mathbf{R}}_1{\mathbf{W}}_{\ast }}+\sqrt{{{\mathbf{W}}_{\ast}}^{\mathrm{T}}{\mathbf{R}}_2{\mathbf{W}}_{\ast }}\right)}\kern0.75em , $$

9 $$ {b}_{\ast }={{\mathbf{W}}_{\ast}}^{\mathrm{T}}{\boldsymbol{\upmu}}_2+{\kappa}_{\ast}\sqrt{{{\mathbf{W}}_{\ast}}^{\mathrm{T}}{\mathbf{R}}_2{\mathbf{W}}_{\ast }}={{\mathbf{W}}_{\ast}}^{\mathrm{T}}{\boldsymbol{\upmu}}_1-{\kappa}_{\ast}\sqrt{{{\mathbf{W}}_{\ast}}^{\mathrm{T}}{\mathbf{R}}_1{\mathbf{W}}_{\ast }}\kern0.5em . $$

### Prediction process

**Fig. 9:**
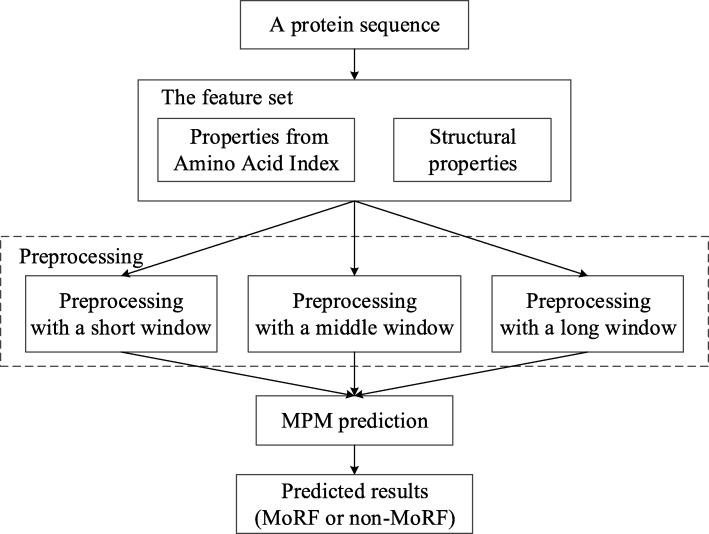
Specific prediction process. Based on the selected feature set, the protein sequence is preprocessed by three different windows, and then is predicted by MPM

For a protein sequence to be predicted, the specific prediction process is shown in the Fig. 9. First, the sequence is preprocessed by the selected feature set with three different windows. Then, the calculated feature matrix is input into the trained MPM, and the predicted result is obtained.

## Data Availability

The datasets supporting the conclusions of this article are available on the references [14, 28].

## Abbreviations

AA Index: Amino Acid Index
IDP: Intrinsically disordered protein
IDR: Intrinsically disordered region
MoRFs: Molecular recognition features
MPM: Minimax probability machine
PDB: Protein Data Bank
PSSM: Position Specific Scoring Matrices
RBF: Radial basis function
SLiMs: Short linear motifs
SVM: Support vector machine
